# Evolution of weak cooperative interactions for biological specificity

**DOI:** 10.1073/pnas.1815912115

**Published:** 2018-11-07

**Authors:** Ang Gao, Krishna Shrinivas, Paul Lepeudry, Hiroshi I. Suzuki, Phillip A. Sharp, Arup K. Chakraborty

**Affiliations:** ^a^Department of Chemical Engineering, Massachusetts Institute of Technology, Cambridge, MA 02139;; ^b^Department of Physics, Massachusetts Institute of Technology, Cambridge, MA 02139;; ^c^Institute for Medical Engineering & Science, Massachusetts Institute of Technology, Cambridge, MA 02139;; ^d^Ragon Institute of Massachusetts General Hospital, Massachusetts Institute of Technology and Harvard University, Cambridge, MA 02139;; ^e^Koch Institute for Integrative Cancer Research, Massachusetts Institute of Technology, Cambridge, MA 02139;; ^f^Department of Biology, Massachusetts Institute of Technology, Cambridge, MA 02139;; ^g^Department of Chemistry, Massachusetts Institute of Technology, Cambridge, MA 02139

**Keywords:** weak cooperative interactions, specificity, evolvability, gene regulation, phase separation

## Abstract

A hallmark of biological systems is that particular functions and outcomes are realized in specific contexts, such as when particular signals are received. One mechanism for mediating specificity is described by Fisher’s “lock and key” metaphor, exemplified by enzymes that bind selectively to a particular substrate via specific finely tuned interactions. Another mechanism, more prevalent in multicellular organisms, relies on multivalent weak cooperative interactions. Its importance has recently been illustrated by the recognition that liquid-liquid phase transitions underlie the formation of membraneless condensates that perform specific cellular functions. Based on computer simulations of an evolutionary model, we report that the latter mechanism likely became evolutionarily prominent when a large number of tasks had to be performed specifically for organisms to function properly. We find that the emergence of weak cooperative interactions for mediating specificity results in organisms that can evolve to accomplish new tasks with fewer, and likely less lethal, mutations. We argue that this makes the system more capable of undergoing evolutionary changes robustly, and thus this mechanism has been repeatedly positively selected in increasingly complex organisms. Specificity mediated by weak cooperative interactions results in some useful cross-reactivity for related tasks, but at the same time increases susceptibility to misregulation that might lead to pathologies.

## Significance

Functional specificity in biology is mediated by two classes of mechanisms, “lock–key” interactions and multivalent weak cooperative interactions (WCI). Despite growing evidence that WCI are widely prevalent in higher organisms, little is known about the selection forces that drove its evolution and repeated positive selection for mediating biological specificity in metazoa. We report that multivalent WCI for mediating biological specificity evolved as the number of tasks that organisms had to perform with functional specificity became large (e.g., multicellular organisms). We find that the evolution of multivalent WCI confer enhanced and robust evolvability to organisms, and thus it has been repeatedly positively selected. Thus, we provide insights on the evolution of WCI and, more broadly, on the evolution of evolvability.

Living organisms have evolved to perform diverse tasks with functional specificity using different mechanisms (1, 2). Unlike highly specific enzymes that have structured recognition domains, many proteins have intrinsically disordered regions (IDRs) that do not fold into ordered structures (3). These proteins often mediate specific biological outcomes through multivalent weak cooperative interactions (WCI). For example, the highly disordered protein histone H1 binds to its chaperone prothymosin-α with specificity (4) to enable chaperone function. This specificity is obtained not through structured “lock and key” interactions, but through multiple cooperative interactions based on coarse-grained associations of short tracks of amino acids of certain lengths and charge patterns, and lack of aromatic side chains. Many cytoplasmic proteins contain multiple recognizable domains (such as SH2 and SH3), which contain low-affinity motifs in disordered backgrounds, which regulate specific biological outcomes via multivalent WCI (3, 5). Proteins with IDRs that interact through such interactions are common in liquid-like condensates (6–8) that form in the cytoplasm and the nucleus to mediate specific biological functions by compartmentalizing particular biochemical pathways.

The most common and rapidly evolving molecular feature of biological systems is changes in gene regulation. In prokaryotes, transcription is regulated by proteins that bind to promoters with high sequence specificity. In mammalian cells, activation of RNA Pol II at the transcription initiation site frequently depends on the binding of multiple proteins to distal noncoding DNA elements called enhancers. It is widely appreciated that the number of enhancers and their constituents change rapidly during evolution (9) and that this variation is critical for functional and morphological differences. Many enhancer binding proteins exhibit specificity of binding to a particular enhancer because of cooperative interactions with other proteins (10). Approximately 52% of DNA and 44% of RNA binding proteins in humans contain IDRs greater than 50 amino acids in length (nearly twofold more common than in the entire proteome). Many of these have been shown to form liquid-like condensates at high concentrations (7), and at lower concentrations when mixed with RNA (11, 12). Clusters of enhancer elements in close physical proximity, known as super-enhancers, regulate the transcription of genes important for maintaining cell identity (13, 14). Recent evidence (15, 16) suggests that multivalent WCI among transcription factors, coactivators, and other transcriptional machinery result in their accumulation at genes regulated by SEs by forming a phase-separated condensate. Because of the cooperative nature of phase transitions, this phenomenon occurs when upstream signals, valency of interactions, or concentration exceeds a sharp threshold (i.e., with functional specificity).

Specificity mediated by multivalent WCI is more prevalent in organisms that have evolved more recently (1). Examples that highlight this evolutionary trend include the observation that the fraction of the proteome containing IDRs is higher in more recently evolved organisms (17), gene regulation in mammals versus prokaryotes noted above, and pathogen recognition mediated by multivalent WCI in vertebrate adaptive immunity (18). Other examples of WCI can be found in signal transduction pathways, extracellular matrix variation, and various cytoskeletal processes (1).

Despite these observations, little is known about the selection forces that drove the predominance of multivalent WCI in mediating biological specificity in more recently evolved organisms. Here, we develop an easily interpretable model that is applicable to a broad class of biological processes and systems and use it to simulate evolution on a computer. Our results provide important insights into why WCI evolved, why it has been repeatedly selected across metazoa, and more generally on the evolution of evolvability.

## Model Development and Methods

We consider a population of organisms that evolve as the number of tasks that they need to perform to function properly increases. Each organism has a number of genes, and the corresponding gene products can potentially perform the tasks. We ignore epistatic interactions between genes, but different gene products can potentially cooperate to perform functions together as described below.

The tasks that organisms must perform with functional specificity can be quite complex (e.g., gene regulation, stress responses, immune responses), but they are considered to be predicated on protein–protein recognition. Thus, our model is based on interactions between gene products and the tasks that they must perform. In performing a task with functional specificity the protein–protein interaction could have lock–key characteristics or be mediated by multivalent WCI between proteins.

**Fig. 1.**
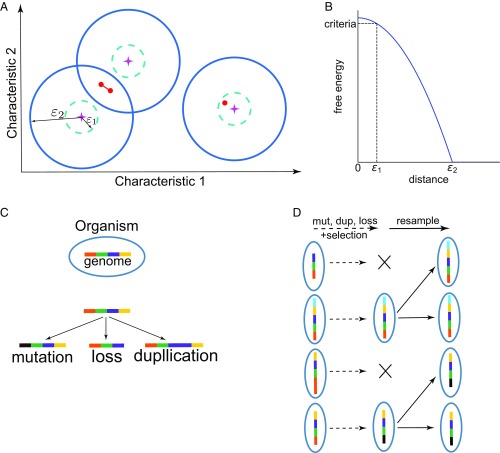
Representation of the evolutionary model. (*A*) A schematic depiction of the space that represents the gene products of organisms and the tasks that they need to perform to function properly. Each axis describes a particular characteristic of a task or matching characteristic in a gene product that determines their interactions (see *Model Development and Methods*). Tasks are shown as stars and gene products as red dots. When a task and a gene product are within a distance equal to ε_1_, the gene product performs the corresponding task with high specificity. When a task and a gene product are within a distance equal to ε_2_, the gene product performs the corresponding task incompletely. When two gene products have closely matched interaction characteristics, they can act cooperatively (indicated with a line connecting them above), to perform tasks together (see *Model Development and Methods*). (*B*) The free energy of interaction between a task and a gene product is defined to be a function of the distance between a task and a single gene product as shown in the graph. The interaction free energy is parabolic when the task-gene distance is less than ε_2_ and becomes 0 when the distance is larger than ε_2_. As defined in *Model Development and Methods*, for cooperating gene products, their free energies with a given task are added up. (*C*) Schematic depiction of the processes of gene mutation, loss, and duplication included in the evolutionary model. For example, in this schematic the orange gene has mutated to black, the yellow gene is lost, and the purple gene is duplicated. (*D*) Depiction of the model for evolutionary dynamics (only one generation of evolution is depicted).

Inspired by models of protein–protein interactions where a few characteristics determine interaction strengths (19), each task and gene product is associated with specific values of a set of characteristics important for their interactions. The value of each relevant characteristic (e.g., hydrophobicity) of a particular task is represented by its position on an axis (Fig. 1*A*). Thus, each task is specified by its positions on different axes that describe each characteristic; i.e., by the position of the task in the space spanned by the axes corresponding to interaction characteristics. For brevity, hereafter we will refer to this space as “characteristic space.” The gene products that perform the tasks are also represented by positions in a characteristic space that describes interaction characteristics that match those that define the tasks. For example, if one axis in the task characteristic space corresponds to hydrophobicity of the tasks, the corresponding axis in the characteristic space in which gene products are represented define the latter’s functional hydrophobicity. Alternatively, if a particular axis in the characteristic space for tasks represents positive charge, the corresponding axis in the characteristic space for gene products represents negative charge. The position of each gene product in its characteristic space is specified by how well matched each of its interaction characteristics is with respect to the characteristics that define the tasks. So, given a set of interaction characteristics defining tasks, there is a known mapping between the task characteristic space and that in which the gene products are represented. Using this mapping, every axis in the gene product characteristic space can be made to coincide with the corresponding axis in the task characteristic space. For example, for charges, the two axes would coincide upon reversing the sign of the axis in the gene product characteristic space. So, in our model, we assume that the mapping has been applied, and thus, the closer a gene product and a task are on the same axis, the more matched they are with respect to the corresponding characteristic, thus contributing to a favorable interaction. Considering all of the characteristics together, the closer a task and a gene product are in characteristic space (Fig. 1*A*), the more favorable their functional interaction.

To construct a general model applicable to diverse examples where WCI have evolved to mediate specificity, we do not specify the particular characteristics that define the tasks. They could be different for each example, and given the way we have defined the model, our results would still be applicable. The number of axes corresponds to the number of characteristics required to describe the protein–protein interactions that predicate tasks being performed. We assume that the number of axes needed is not large, since the strength of protein–protein interactions is usually determined by a small number of key relevant quantities (charge, charge distribution, hydrophobicity). Our qualitative results are insensitive to the particular choice of a finite number of axes (*SI Appendix*, Figs. S9–S11).

The fitness of an organism depends on how well its gene products perform the tasks. If a gene product is within a short distance, ε_1_, of a task (Fig. 1*A*), it is considered to perform this task with functional specificity via strong interactions. If the distance between a task and a gene product is within a larger distance, ε_2_, then the task is considered to be done less completely via weak interactions. If a gene product is located a distance further away from the task than ε_2_, then the interactions are too weak for the task to be done by this gene product.

If two or more gene products are within a short distance, ε_3_, from each other, they can interact with each other and potentially act cooperatively to complete a task with functional specificity although each gene product interacts weakly with the task (i.e., via multivalent WCI). The free energy of interaction between a task and a gene product is considered to be a function of distance as shown in Fig. 1*B*. We model the cooperative action of gene products within a distance ε_3_ from each other by adding up their interaction free energies corresponding to a task (mathematical details in *SI Appendix*, *Supplementary Information Text*). If the resulting number exceeds the value of the free energy corresponding to a distance of ε_1_ for a single gene product interacting with a task, then the gene products are considered to perform the task with functional specificity. Thus, multiple gene products can cooperatively perform a task with specificity via multivalent WCI if their interaction free energies with the task add up to be at least as favorable as that corresponding to a single gene product that performs a task in a lock–key fashion (located within a distance equal to ε_1_ of the task).

Given a set of tasks, we define a function, *F*_*j*_, for organism, *j*, as follows:

$$F_{j}=\lambda_{1}\left(M-\#\;\mathrm{tasks}\;\mathrm{done}\;\mathrm{by}\;j\right)+\lambda_{2}\left(M\;-\;\#\;\mathrm{tasks}\;\mathrm{done}\;\mathrm{with}\;\mathrm{functional}\;\mathrm{specificity}\;\mathrm{by}\;j\right)+\lambda_{3}G^{j},$$ [1]

where *G*^*j*^ is the number of genes in organism, *j*, and *M* is the number of tasks to be performed for proper function. The fitness of organism, *j*, is defined as $f_{j}=e^{-F_{j}}$. The first term in Eq. **1** makes organisms that perform the tasks, at least poorly, have a higher fitness than those that do not. The second term makes organisms that perform more tasks with functional specificity more fit. The third term makes organisms with bigger genomes less fit than their peers. The quantities λ_1_, λ_2_, and λ_3_ represent the relative weights of these three factors, or selection forces, in determining an organism’s fitness.

We initiate the evolutionary dynamics with a single task that must be performed, and each organism in the population has a single gene. The gene product of each organism is assigned to a randomly chosen point in characteristic space. The organisms evolve by mutation, gene duplication, and gene loss (Fig. 1*C*). Recombination is unlikely to affect the qualitative behavior of the model unless the recombination rate is unusually large. We are concerned here with the evolution of WCI as a mechanism for functional specificity, and this mechanism is more prevalent in higher organisms where horizontal gene transfer is less important. Therefore, we also do not consider horizontal gene transfer.

The organisms evolve according to standard Wright–Fisher evolutionary dynamics with a fixed number of organisms, *N*, in the population (Fig. 1*D*). At each time step, the genome of every organism can potentially undergo mutation, gene duplication, and gene loss. When a gene mutates, the location of its gene product in characteristic space is changed by translating it in a randomly chosen direction by a random distance whose average value is ε_1_. The mutation rate is chosen such that, on average, in every organism, one gene is likely to mutate every two time steps. Gene duplication occurs at one-tenth the rate of mutation. A duplicated gene makes a gene product that occupies exactly the same location in characteristic space as its copy. With time, the two genes, and hence their gene products, can diverge from each other and potentially perform different tasks (or functions). Gene loss occurs at the same rate as duplication. After mutation, gene duplication and loss are attempted with the probabilities specified above, each organism can acquire a potentially new genome, with new coordinates in characteristic space for its gene products (Fig. 1*D*).

The probability that an organism will produce a progeny (or be positively selected) in this evolutionary time step is then calculated as follows:

$$\begin{array}{c} p_{s}^{j}=\frac{f_{j}}{\sum_{j=1}^{N}f_{j}}, \end{array}$$ [2]

where $p_{s}^{j}$ is the probability that organism *j* will be present in the next time step of evolution. After this selection step, the number of organisms that produce a progeny is likely to be less than *N* because some organisms die without producing progeny as they are not sufficiently fit. To keep the population size constant as per Wright–Fisher dynamics, we rescale the total number of organisms to remain equal to *N* when the next time step begins (Fig. 1*D*). The proportion of organisms with a particular genome is kept the same as before rescaling (i.e., after selection). This evolutionary process continues in subsequent time steps. The stochastic processes described above are simulated using a Monte Carlo computational procedure.

We characterize the system using the following variables: (*i*) the average number of genes in an organism in the population; (*ii*) the number of tasks completed with functional specificity by an organism via WCI involving multiple gene products, averaged across the population; (*iii*) the number of tasks completed with functional specificity by single gene products in an organism via lock–key interactions, averaged across the population. We carry out the evolutionary dynamics until a “steady state” is reached with respect to these variables; i.e., the system ceases to evolve further because a fitness peak has been reached (*SI Appendix*, Fig. S1). We then introduce a new task in characteristic space (the value of *M* increases by one in Eq. **1**) and carry out the evolutionary dynamics again until steady state, starting from the state of the organisms that were evolutionary fit for the previous tasks. Thus, the evolutionary history of the organisms is explicitly incorporated. This process is repeated as new tasks are introduced. Thus, we study whether, and why, mechanisms for regulating specificity evolve as organisms have to perform more tasks specifically to function properly (e.g., as multicellular organisms became more complex). An important variable is the extent to which the newly introduced task is correlated, or similar, to the existing tasks. We have studied several cases that are described in *Results*.

The parameters in the model are ε_1_, ε_2_, ε_3_, λ_1_, λ_2_, λ_3_, and the extent to which newly introduced tasks are correlated with the existing ones. Based on parameter sensitivity studies (*SI Appendix*, Figs. S2–S7), we note that the qualitative results that we report are robust as long as λ_1_ and λ_2_ are greater than λ_3_. If λ_3_ becomes too large, the introduction of new genes leads to severe fitness penalties. So, when the number of tasks that must be performed for proper function becomes large, the organisms prefer to have reduced fitness by not functioning properly (i.e., not completing the necessary tasks) rather than evolve new genes. This is tantamount to being unable to evolve more complex multicellular organisms, and so we do not consider this case further. The values of the parameters used to obtain the results discussed below are ε_1_ = ε_3_ (which equals the size of a single mutation step in our model), ε_2_ = 5ε_1_, λ_1_ = λ_2_ = 1, and λ_3_ = 0.1. The dependence of the results on changing the value of ε_3_ and λ_1_ will be discussed below. Choosing ε_1_ to be the same as the size of a single mutation implies that the condition for functional specificity via lock–key fit is stringent.

## Results

**Fig. 2.**
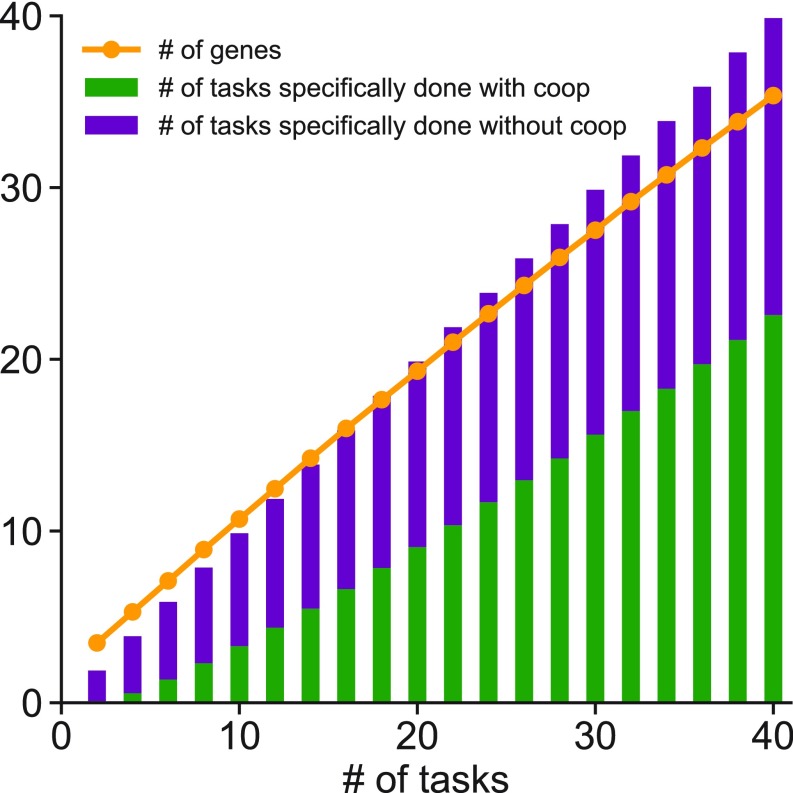
WCI evolve as organisms become more complex. This figure shows the variation of the average number of genes in organisms and the number of tasks specifically done via WCI between gene products as the number of tasks required for an organism to function properly increases (or organisms become more complex). The number of tasks performed by single gene products is also shown. When the number of tasks equals 10, 33% of tasks are done via WCI, and when the number of tasks equals 40, this proportion is 56%. Three characteristics describe the interaction characteristics of tasks and gene products.

We first studied a situation wherein each new task is introduced at a randomly chosen location in characteristic space that is at a distance equal to 1.8ε_2_ away from any one of the tasks that had to be previously performed. So, in terms of its interaction characteristics, the newly introduced task has some similarity with previous tasks. Our simulation results (Fig. 2) show that WCI evolve as a mechanism for mediating functional specificity as the number of tasks that organisms have to perform to function properly increases (or organisms become more complex). Furthermore, as organisms evolve to perform more tasks, the proportion of the tasks that they carry out via WCI increases (Fig. 2). These results are consistent with the observation that this mechanism for mediating functional specificity is prevalent in multicellular organisms. One reason that WCI evolved as a mechanism for biological specificity is because this allows similar tasks to be performed with some of the same cooperating components, and therefore, the number of genes required for organisms to function properly becomes less than the number of tasks to be performed (Fig. 2). This is consistent with the observation that proteins with similar IDRs (and even the same proteins) are involved in regulating different genes and in forming condensates at different super-enhancers. The same is true for components that form condensates to mediate other biological functions in the cytoplasm and the nucleus. We have carried out calculations with different levels of correlation between new and old tasks (i.e., values of task-task distance other than 1.8ε_2_), and the qualitative behavior of our model is unchanged (*SI Appendix*, Fig. S4) unless the new tasks become totally uncorrelated.

**Fig. 3.**
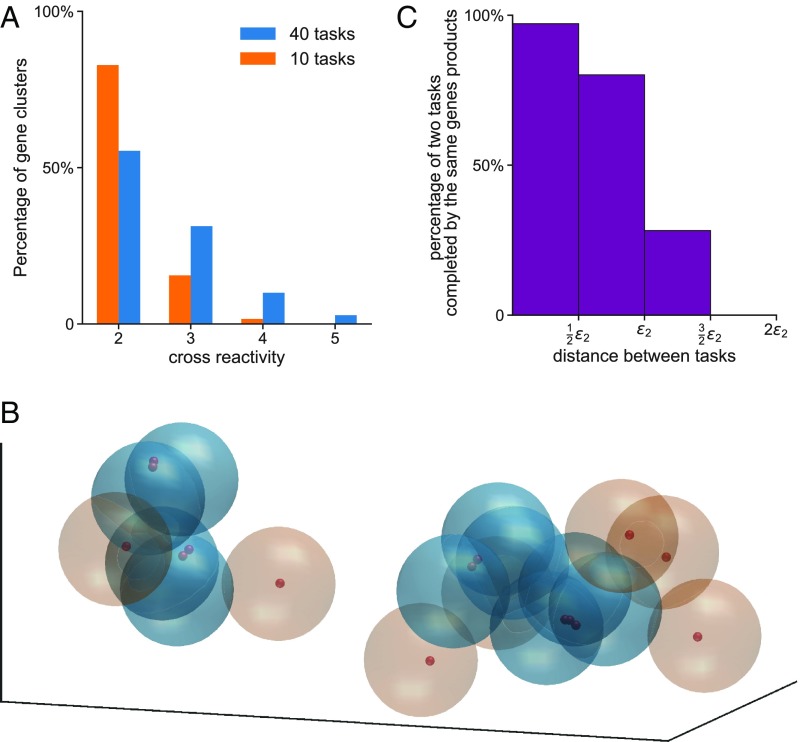
Limited cross-reactivity accompanies the evolution of WCI. (*A*) Variation of the extent of cross-reactivity with the evolution of WCI. The *x* axis shows the number of tasks done by the same cluster of gene products, and the *y* axis is the percentage of such clusters that are preforming two, three, four, and five tasks in this cross-reactive fashion. (*B*) Snapshot of simulation results when new tasks are introduced such that they are either closely related to tasks from an earlier era or not. Two modules of such related tasks are depicted in characteristics space. Large spheres with radius ε_2_ are drawn around each task. Brown spheres show tasks being performed by single-gene products, blue spheres show closely related tasks being performed by clusters of cooperating gene products. Small spheres correspond to gene products. (*C*) The percentage of two tasks completed by the same gene products is high only for related tasks. Three characteristics describe the interaction characteristics of tasks and gene products.

One implication of the results described so far is that as a greater proportion of tasks are performed via WCI (as the number of tasks increases), the extent to which gene products are cross-reactive to multiple tasks also increases. The results in Fig. 3*A* show that this is indeed the case. However, the cross-reactivity is limited to similar tasks. This can be seen clearly by considering a situation where a newly introduced task can either be closely related to one of the previous tasks or not. If new tasks that are related to at least one previous task are introduced more frequently than tasks that are unrelated (75% chance for a new task to be at a distance 1.8ε_2_ away from a previous task and 25% chance to be at least at a distance 3.0ε_2_ away from all previous tasks), the tasks will be distributed in characteristic space as disjoint groups of related tasks (Fig. 3*B*). One group may correspond to regulation of gene transcription; another could be signaling through SH2/SH3 domains in the cytoplasm. Fig. 3*B* illustrates that gene products that act via WCI are cross-reactive to a limited set of tasks that are closely related. Quantitatively, the number of tasks that are performed by the same gene products acting cooperatively rapidly declines as the interaction characteristics of the tasks become less related (Fig. 3*C*).

Some cross-reactivity for similar tasks is an inherent property of the above cooperative model, but the extent of cross-reactivity is limited as otherwise task specificity would be lost. In cells, other mechanisms can be coupled to multivalent WCI to limit cross-reactivity. For example, master transcription factors bind with lock–key type specificity to particular DNA binding sites. Only then can interactions between transcription factor IDRs and that of transcriptional coactivators, chromatin remodelers, and RNA Polymerase II occur through multivalent WCI if specific upstream signals have modified the IDRs to have a valency exceeding a threshold. However, the coactivators, chromatin remodelers can exhibit some cross-reactivity (as in Fig. 3) to regulate related functions, such as genes bound by different master transcription factors. The degree of cross-reactivity could also be limited by topological barriers such as chromosomal domains or localization in subcellular compartments. However, the cross-reactivity that accompanies the evolution of WCI for biological specificity could, when altered by mutation or modification, cause serious pathologies. For example, protooncogenes can be activated when DNA rearrangements create a fusion protein that targets transcriptional activation domains in their vicinity (20, 21). Also, cellular states that generate abnormally large condensates (22) formed by multivalent WCI could sequester high levels of client proteins important for the normal functioning of other genes.

**Fig. 4.**
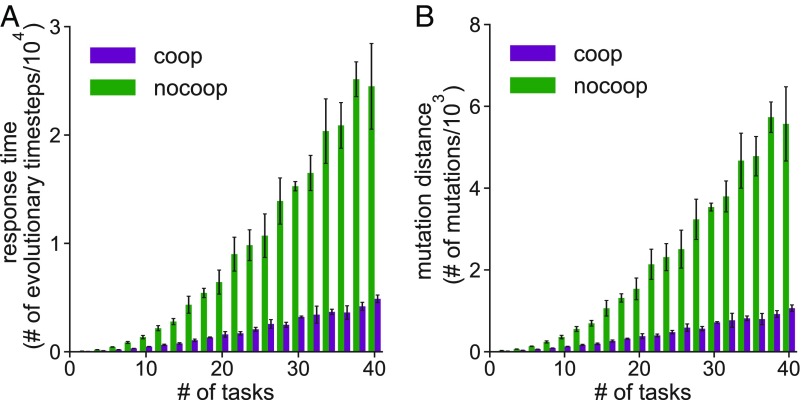
The evolution of WCI for biological specificity makes organisms more evolvable. (*A*) The response time for the organisms to evolve to function properly after a new task is introduced is shown as a function of the number of tasks (or complexity). Results are shown for both the full model and one wherein cooperative interactions between gene products is not allowed. (*B*) The number of mutations (which includes gene mutation, duplication, and loss) that the average organism needs to acquire to function properly after a new task is introduced is shown as a function of the number of tasks (or complexity). Results are shown for both the full model and one wherein cooperative interactions between gene products is not allowed.

Multivalent WCI as a mechanism underlying biological specificity are prevalent in many organisms across metazoa. We wondered whether the emergence of this mechanism makes organisms more evolvable, thus explaining why it has been repeatedly positively selected and its more prominent role in multicellular organisms. The properties of more evolvable systems (1) include the following: (*i*) Reduced constraints in maintaining old functions when a new function has to be evolved and (*ii*) fewer mutations required to produce novel phenotypic traits. Thus, to explore this question, we calculated the time required for organisms to evolve to perform the tasks required for proper function after a new task is introduced. We compared the results of simulations of our model to one where WCI are not allowed; i.e., regardless of the similarity between the interaction characteristics of gene products, they are not allowed to act in concert to perform tasks with functional specificity. As Fig. 4 shows, the model allowing the evolution of WCI exhibits shorter response times and requires fewer mutations to respond to new tasks. Increasing values of ε_3_ also make mutations increasingly less likely to be lethal when WCI are allowed (*SI Appendix*, Fig. S8). This is because a deleterious mutation in any one gene product involved in mediating functional specificity via multivalent WCI is less likely to result in loss of function (task not performed) compared with the effect of a similar mutation for lock–key interactions. We conclude that the evolution of WCI for functional specificity confers increased and robust evolvability to organisms, as they can evolve to perform new tasks while maintaining old functions with fewer mutations and increased tolerance to deleterious mutations. This result is reinforced by a recent report demonstrating rapid evolution of human IDR proteins (23). Notice that evolvability emerges in our model without violating causality, i.e., this mechanism evolves based on past selection forces and not a pathological knowledge of the future.

The qualitative results that we have described hold if there is no fitness advantage associated with an organism performing a task poorly (λ_1_ equals zero in Eq. **1**). The only difference is that the response times for organisms to evolve to perform new tasks increase (*SI Appendix*, Fig. S3). That is, the system becomes less evolvable if there is no fitness advantage for performing tasks poorly. This is because the lack of ability to be positively selected while performing tasks poorly constrains the mutational trajectories that have to be followed to perform new tasks while not abrogating old functions. A similar observation has been made in laboratory experiments following the mutational pathway of a kinase as it evolves to catalyze a new substrate (24). Mutations are first observed in the kinase’s allosteric pocket resulting in conformational flexibility that enables it to act on the old and new substrates suboptimally. Then, a mutation in the catalytic site is acquired to change specificity. A similar effect has also been described during the evolution of cross-reactive antibodies during germinal center reactions (25).

## Discussion

Biological systems carry out tasks with functional specificity. We have considered a model where the ability of an organism to carry out tasks is predicated on protein–protein recognition mediated by either lock–key or multivalent WCI. The ability of an organism to function properly, or its fitness, depends upon whether it can carry out a set of tasks with functional specificity (described by the first two terms in Eq. **1**). We simulated the evolution of a population of such organisms as the number of tasks that need to be carried out for organisms to function properly progressively increases (larger values of *M*). The fitness landscape, or the genotype–phenotype relationship, changes as *M* increases. Thus, the organisms mutate to try to achieve a phenotype that is more fit—viz., a phenotype that can carry out the larger number of required tasks with functional specificity. Our results show that WCI emerge as a prominent mechanism for mediating specificity as organisms have to carry out larger number of tasks with specificity. We argue that this is the reason that WCI are more prominent in multicellular organisms. The evolution of WCI as a mechanism for mediating specific biological functions allows higher organisms to carry out diverse tasks with a relatively small genome (in our model genome size is constrained by the third term in Eq. **1**).

Our model also shows that the emergence of WCI makes organisms more evolvable in that, as new tasks are introduced, the population of organisms can evolve to higher fitness phenotypes faster and with fewer mutations. Furthermore, as organisms mutate to try and acquire higher fitness, mutations are less likely to be lethal after WCI emerge as a mechanism that mediates functional specificity. In other words, the fitness landscape describing the genotype–phenotype relationship becomes less rugged when WCI evolve. It has been noted (1, 2) that the more exact or precise the requirements for function, the less evolvable the system is. Performing tasks with functional specificity mediated by WCI does not require the level of biochemical precision characteristic of lock–key interactions, and thus the system becomes more evolvable. We argue that WCI have been repeatedly positively selected in higher organisms because of the enhanced evolvability conferred by this mechanism for functional specificity. Indeed, the evolution of WCI may have given metazoans the great capacity for change whose consequences we observe today.

Our model is consistent with the observation that weak interactions have evolved to be highly relevant for gene regulation in metazoa. The IDRs of transcription factors and coactivators leverage WCI to drive condensate formation at regulatory elements to mediate transcription in higher organisms. This is in contrast to prokaryotes, where gene regulation is largely dictated by lock–key interactions that promote localization of TFs to specific promoter sequences. The biochemical rules for the WCI that determine interactions between IDRs is not as precise as specific enzyme–substrate interactions. Thus, the same IDRs can be employed to perform related functions, and IDRs can evolve readily with few mutations to regulate new functions. Thus, these motifs have been conserved in higher organisms. In the future it will be interesting to see the molecular grammar that determines WCI in these contexts.

A much higher fraction of proteins (17) in multicellular organisms, compared with prokaryotes, possess IDRs, and these IDRs are strongly enriched in factors controlling regulatory processes. These IDR regions, frequently in combination with RNA and/or DNA binding, provide some of the valency necessary to form condensates and concentrate factors in regulatory pathways (11, 12). Recent analysis (23) of the rate of evolutionary change in the IDR regions suggests that they are more tolerant of mutational variation than regions with structured domains but are nevertheless under genetic constraint. Since regulatory variation is thought to be the most rapidly changing aspect of evolutionary change in multicellular organisms, it is perhaps not surprising that WCI are concentrated in these networks (26).

The same type of reasoning probably explains the common observation that many regulatory RNA binding proteins in multicellular organisms possess limited sequence specificity (three to four nucleotides), while the total sequence complexity of expressed coding and noncoding RNAs in cells is enormous. Similar examples can be found in signal transduction pathways, extracellular matrix variation, and various cytoskeletal elements (1). In their discussions about evolvability and facilitated variation (1, 2), Kirschner and Gerhart anticipated WCI as an important aspect of multicellular biology, proposing that “weak linkage,” compartmentalization, and redundancy contribute to constraint reduction, thus resulting in the robustness and observed regulatory variability in these organisms. Our model predicts the evolution of these characteristics (i.e., WCI) in organisms when they are challenged with new tasks, under constraints that limit the unbounded growth of the number of genes. Simply stated, these features—and evolvability—emerge organically from the known physical structures and interactions of proteins, RNA, and DNA on which the model is based.

This model describes many types of specific biological functions beyond gene regulation. For example, unlike more ancient organisms, vertebrates have an adaptive immune system that can mount pathogen-specific responses against a diverse and evolving world of microbes (18). The immune system is routinely faced with performing new tasks (recognize foreign pathogens not encountered previously) with functional specificity. One way it achieves this goal is to generate diverse receptors of B and T lymphocytes that interact with pathogenic markers. Importantly, functional specificity for particular pathogenic markers is achieved by the receptors via multivalent WCI (19, 27–29). The receptor on a particular lymphocyte commonly exhibits cross-reactivity to a few pathogen-derived ligands (30). Some degree of cross-reactivity helps with recognizing a vast space of antigens, but pathogen specificity requires that responses are not too broadly cross-reactive. This limited cross-reactivity naturally emerges from our model as cross-reactivity is limited to similar tasks.

Many studies have considered the evolution of modularity when there is a frequently changing environment (31–34). Modules are units with highly interconnected moieties that interact with other modules via very few interactions. Modularity make biological systems more evolvable (35) because the modules can be combined with each other differently to carry out new functions, much like subroutines in computer programs can be reused for different computations. Our focus has been on multivalent WCI where the participating components interact with each other via numerous weak interactions to mediate functional specificity while making organisms more evolvable.

The need to efficiently perform new tasks while retaining the ability to functionally execute previously learned tasks is common in many biological systems. For example, this is characteristic of learning by the nervous system. It may also be a characteristic of how computational machine learning algorithms trained on large datasets to predict specific outcomes could be adapted to predict new outcomes. We suspect that the fundamental aspects of the model we have described may be relevant to these situations as well.

## Materials and Methods

The computer code used to generate the results will be made available upon request.

## Supplementary Material

Supplementary File
