# Supplementary material for: Evolution of weak cooperative interactions for biological specificity
Source: Proc Natl Acad Sci U S A. 2018 Nov 7;115(47):E11053–60. doi: 10.1073/pnas.1815912115 (PMC6255166; doi:10.1073/pnas.1815912115)
Supplement: Supplementary File [file pnas.1815912115.sapp.pdf]

PNAS
www.pnas.org

Supplementary Information for

Evolution of weak cooperative interactions for biological specificity

Ang Gao[1], Krishna Shrinivas[1], Paul Lepeudry, Hiroshi I. Suzuki, Phillip A. Sharp[2] and Arup K. Chakraborty[2]

[1]These authors contribute equally.
[2]Correspondence: arupc@mit.edu (A.K.C.), sharppa@mit.edu (P.A.S)

This PDF file includes:

Supplementary Text
Figs. S1 to S11

www.pnas.org/cgi/doi/10.1073/pnas.1815912115

Supplementary Information Text

Mathematical definition of the cooperation of gene products. We use $E(r)$ to denote the free energy of interaction between a task and a gene product. We use $1, \dots, k$ to denote the gene products, and $r_1, \dots, r_k$ to denote the distance between these gene products and a given task. This task is performed specifically by the cooperation of these gene products if $\sum_{i=1}^{k} E(r_i) \geq E(\varepsilon_1)$ and if for each gene product i there exists a gene product j such that $\left|\vec{r}_i - \vec{r}_j\right| < \varepsilon_3$. The definition of ε_1 and ε_3 can be found in Fig. 1A.

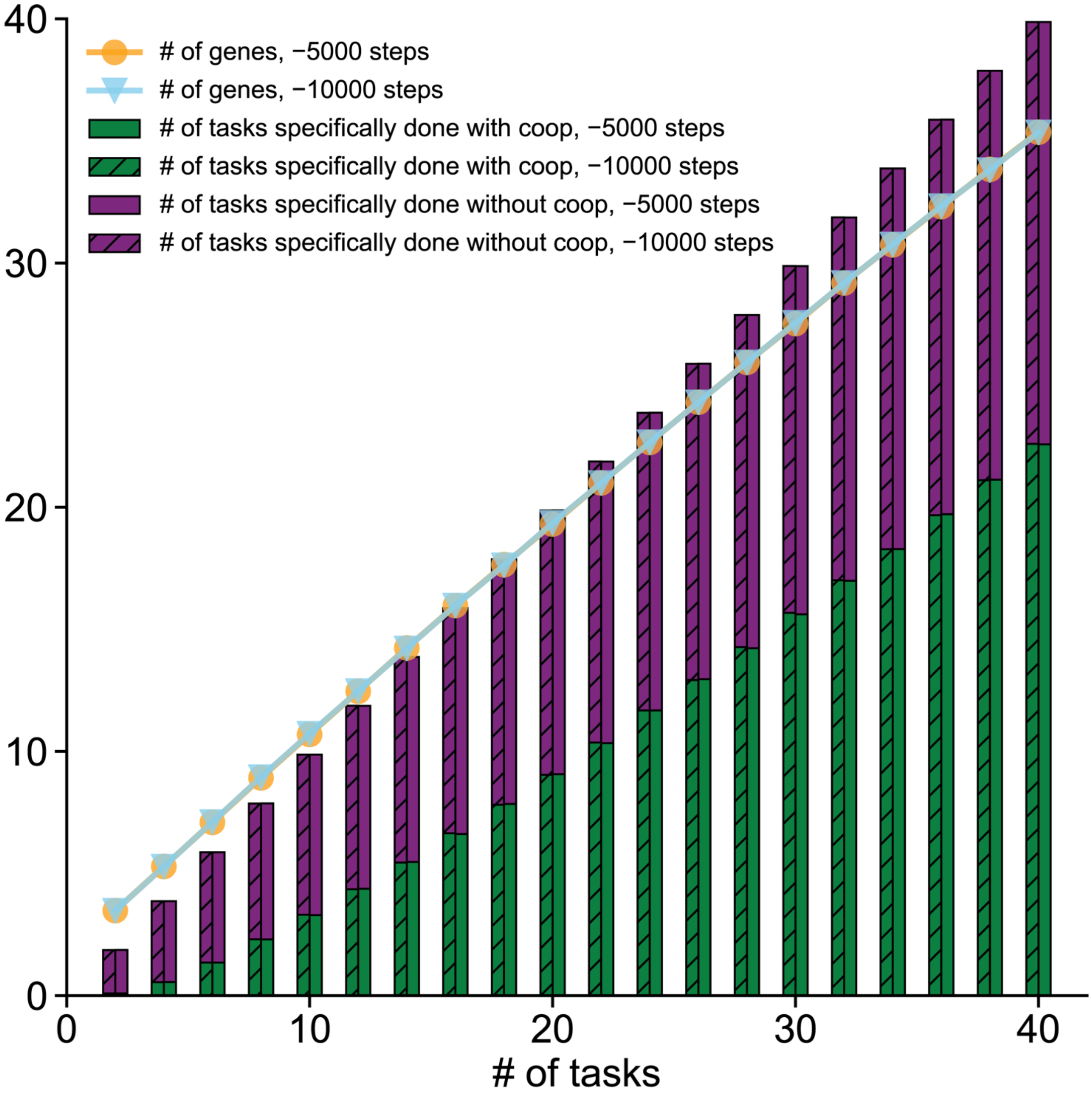

Fig. S1. Steady state is reached before introducing new tasks. Key variables (the number of genes and the number of tasks done with/without weak cooperation of gene products) of the reference system are measured at 5000 steps and at 10000 steps before each new task is introduced, and the results are almost exactly the same, which indicates that the steady state is reached before new task is introduced. The reference system is the one we discussed in the main text, where $\varepsilon_2 = 5\varepsilon_1 = 5\varepsilon_3,\ \lambda_1 = \lambda_2 = 1, \lambda_3 = 0.1,$ the new task is located at distance $1.8\varepsilon_2$ away from one of the previous tasks, and three characteristics are used to describe the interaction characteristics between tasks and gene products.

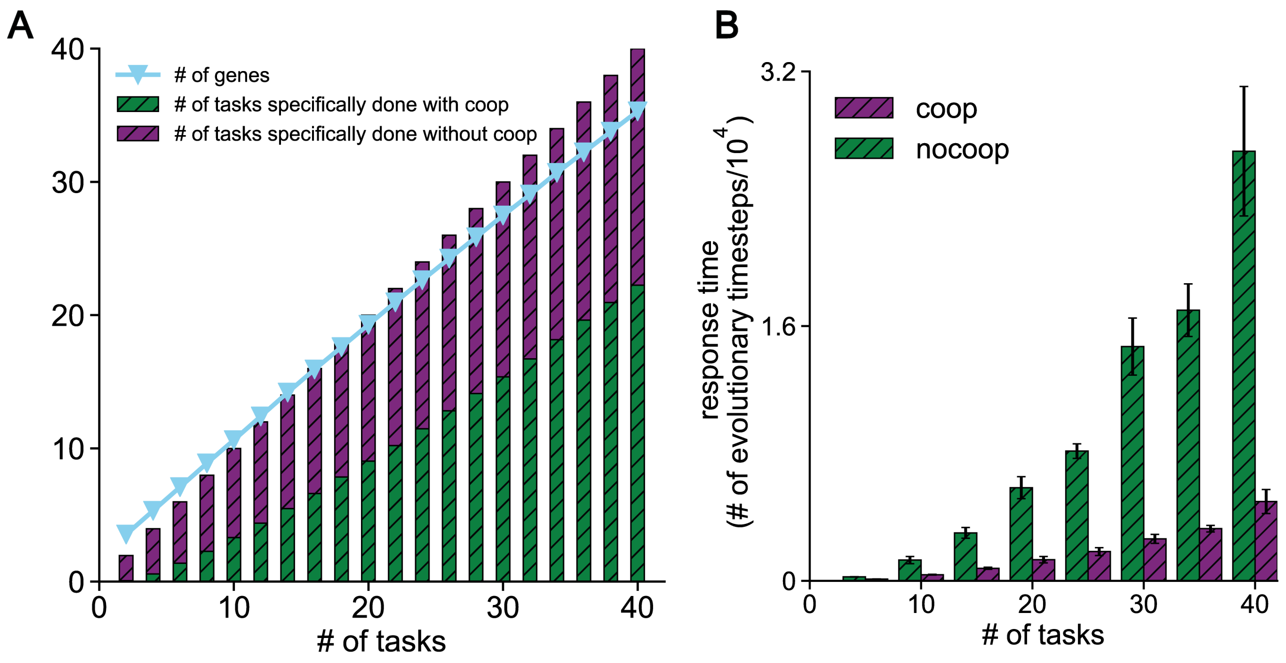

Fig. S2. Simulation results of the system where λ_1 and λ_2 is changed to 10. The other parameters of this system are kept the same as those of the "reference system". The reference system is the one we discussed in the main text, where $\varepsilon_2 = 5\varepsilon_1 = 5\varepsilon_3,\ \lambda_1 = \lambda_2 = 1, \lambda_3 = 0.1$, the new task is located at distance $1.8\varepsilon_2$ away from one of the previous tasks, and three characteristics are used to describe the interaction characteristics between tasks and gene products.
(A) Variation of the number of genes and the number of tasks specifically done with/without weak cooperation of gene products as the number of tasks required for an organism to function properly increases.
(B) The response time for the organisms to evolve to function properly after a new task is introduced is shown as a function of the number of tasks (or complexity). Results are shown for both the system where λ_1 and λ_2 is changed to 10 and its corresponding "nocoop system" wherein cooperative interactions between gene products are not allowed.

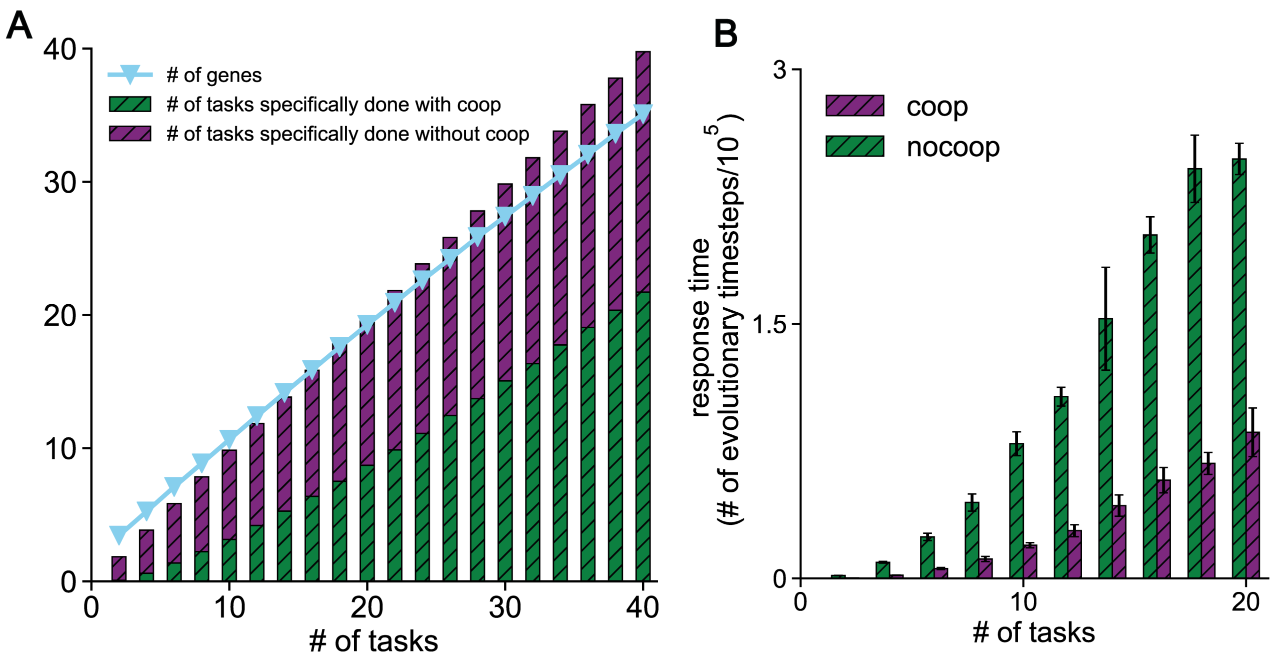

Fig. S3. Simulation results of the system where λ_1 is changed to 0. The other parameters of this system are kept the same as those of the reference system.

(A) Variation of the number of genes and the number of tasks specifically done with/without weak cooperation of gene products as the number of tasks required for an organism to function properly increases.

(B) The response time for the organisms to evolve to function properly after a new task is introduced is shown as a function of the number of tasks (or complexity). Results are shown for both the system where λ_1 is changed to 0 and its corresponding "nocoop system" wherein cooperative interactions between gene products are not allowed. The response time of this "$\lambda_1 = 0$" system is much larger than the response time of the reference system. For example, the response time for this "$\lambda_1 = 0$" system to complete the 20th task is 86000, while the response time for the reference system to complete the 20th task is 4900.

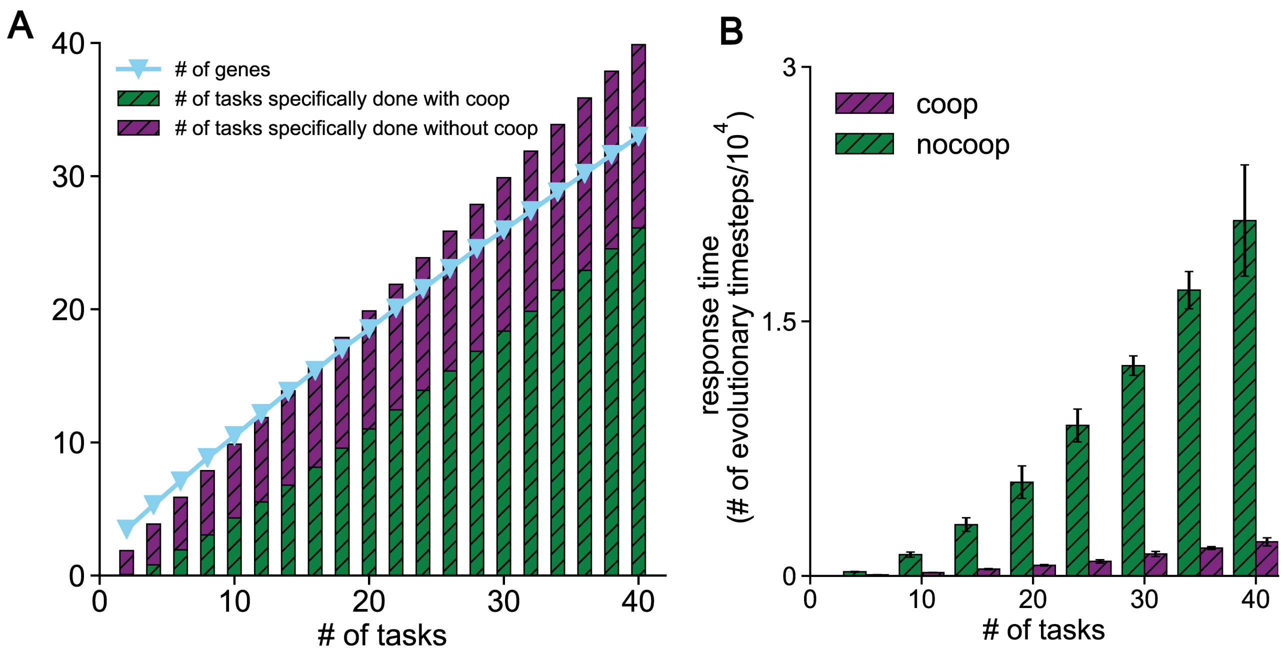

Fig. S4: Simulation results of the system where correlation between tasks are increased (the new task is located at distance $1.5\varepsilon_2$ away from one of the previous tasks.). The other parameters of this system are the same as those of the reference system.
(A) Variation of the number of genes and the number of tasks specifically done with/without weak cooperation of gene products as the number of tasks required for an organism to function properly increases.
(B) The response time for the organisms to evolve to function properly after a new task is introduced is shown as a function of the number of tasks (or complexity). Results are shown for both the system which has increased correlation between tasks and its corresponding "nocoop system" wherein cooperative interactions between gene products are not allowed.

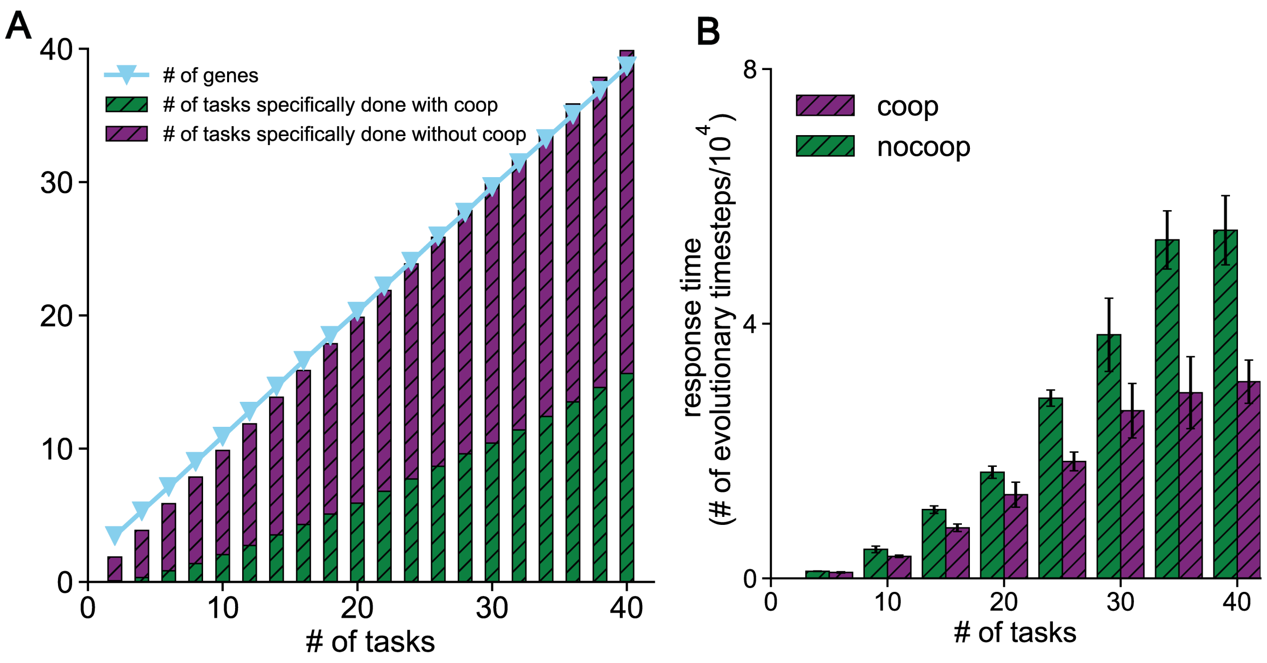

Fig. S5 Simulation results of the system where correlation between tasks are decreased (the new task is located at distance $2.5\varepsilon_2$ away from one of the previous tasks.). The other parameters are the same as those of the reference system.
(A) Variation of the number of genes and the number of tasks specifically done with/without weak cooperation of gene products as the number of tasks required for an organism to function properly increases.
(B) The response time for the organisms to evolve to function properly after a new task is introduced is shown as a function of the number of tasks (or complexity). Results are shown for both the system which has decreased correlation between tasks and its corresponding “nocoop system” wherein cooperative interactions between gene products are not allowed.

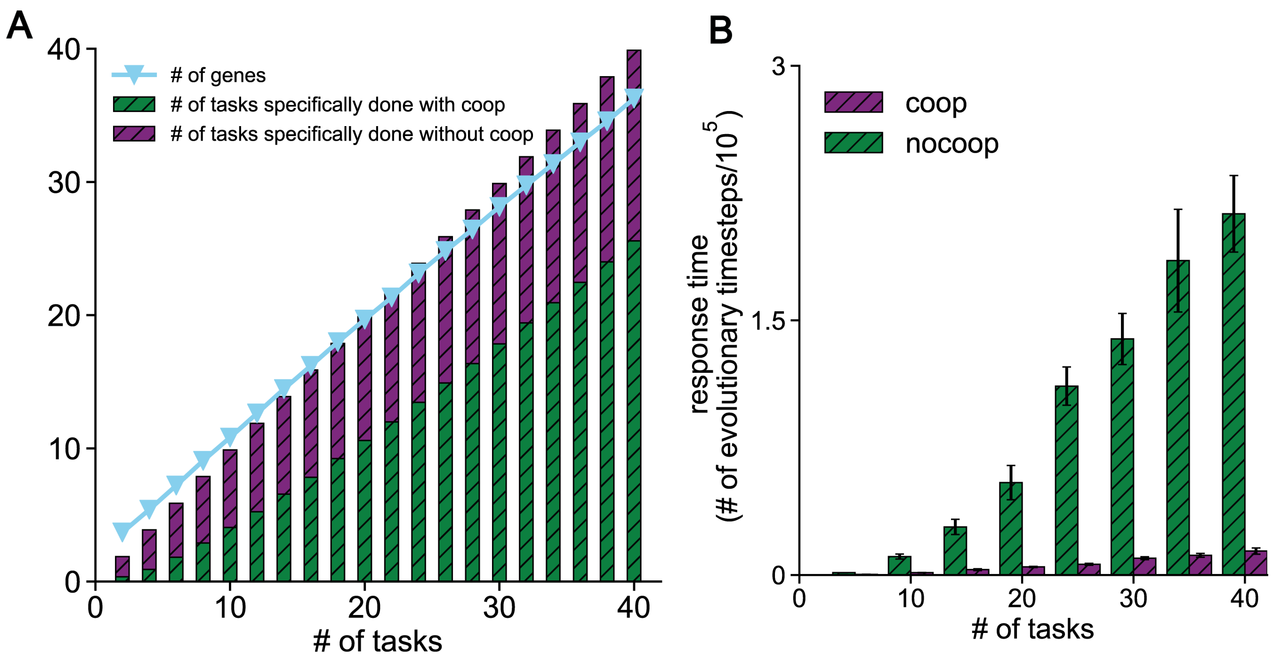

Fig. S6. Simulation results of the system where ε_1 is changed to $\frac{5}{8}$ that of the reference system. The other parameters of this system are the same as those of the reference system.
(A) Variation of the number of genes and the number of tasks specifically done with/without weak cooperation of gene products as the number of tasks required for an organism to function properly increases.
(B) The response time for the organisms to evolve to function properly after a new task is introduced is shown as a function of the number of tasks (or complexity). Results are shown for both the system where ε_1 is changed to $\frac{5}{8}$ that of the reference system and its corresponding "nocoop system" wherein cooperative interactions between gene products are not allowed.

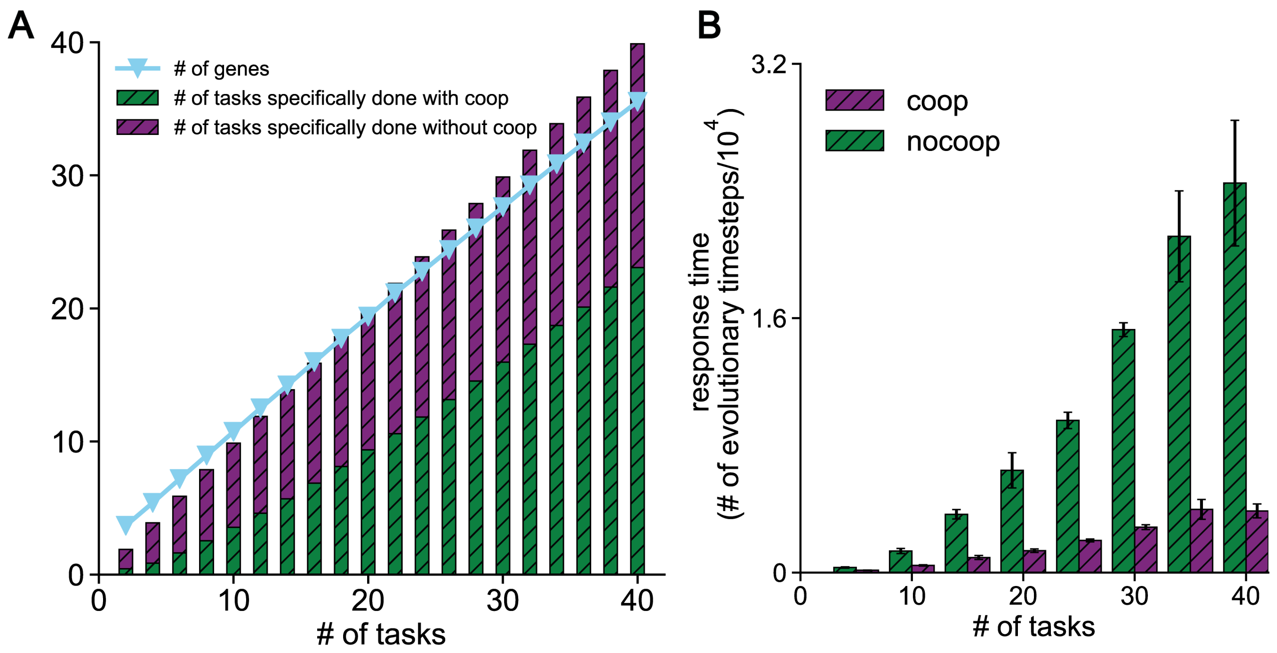

Fig. S7: Simulation results of the system where ε_3 is changed to twice that of the reference system. The other parameters of this system are the same as those of the reference system.
(A) Variation of the number of genes and the number of tasks specifically done with/without weak cooperation of gene products as the number of tasks required for an organism to function properly increases.
(B) The response time for the organisms to evolve to function properly after a new task is introduced is shown as a function of the number of tasks (or complexity). Results are shown for both the system where ε_3 is changed to twice that of the reference system and its corresponding "nocoop system" wherein cooperative interactions between gene products are not allowed.

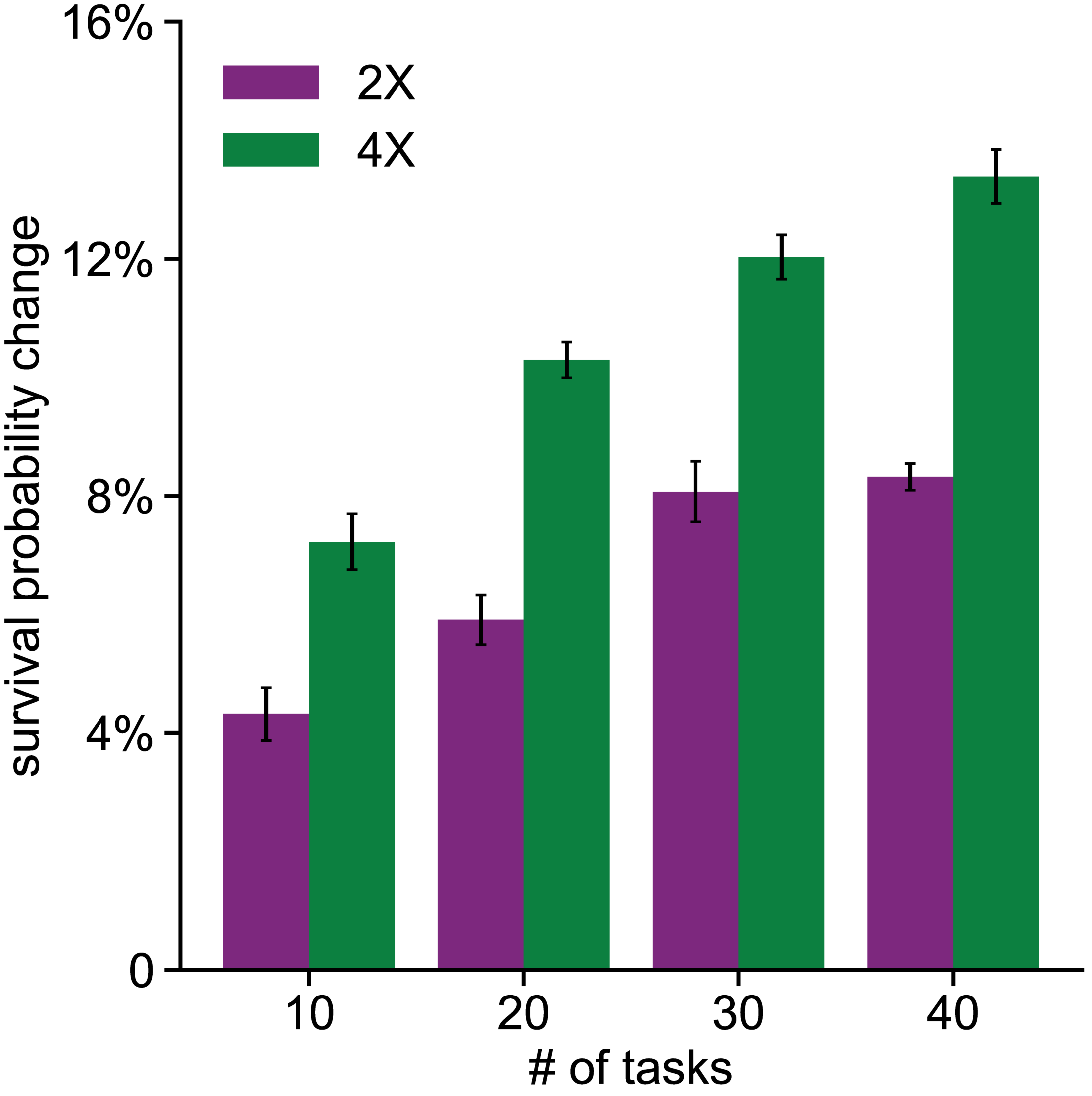

Fig. S8. The survival probability of mutated organisms is increased when ε_3 is increased. The survival probability of mutated organisms is the probability that the mutated organisms will be present in the next time step of evolution. The change of survival probability when ε_3 becomes twice that of the reference system as a function of the number of tasks that the organisms need to perform is shown as the purple bars. The change of survival probability when ε_3 becomes four times that of the reference system as a function of the number of tasks that the organisms need to perform is shown as the green bars. Further simulation results show that the survival probability of the mutated organism becomes larger than that of the corresponding "nocoop system" when ε_3 is more than twice larger than that of the reference system (i.e., when ε_3 is more than twice larger than the stepsize of the gene mutation).

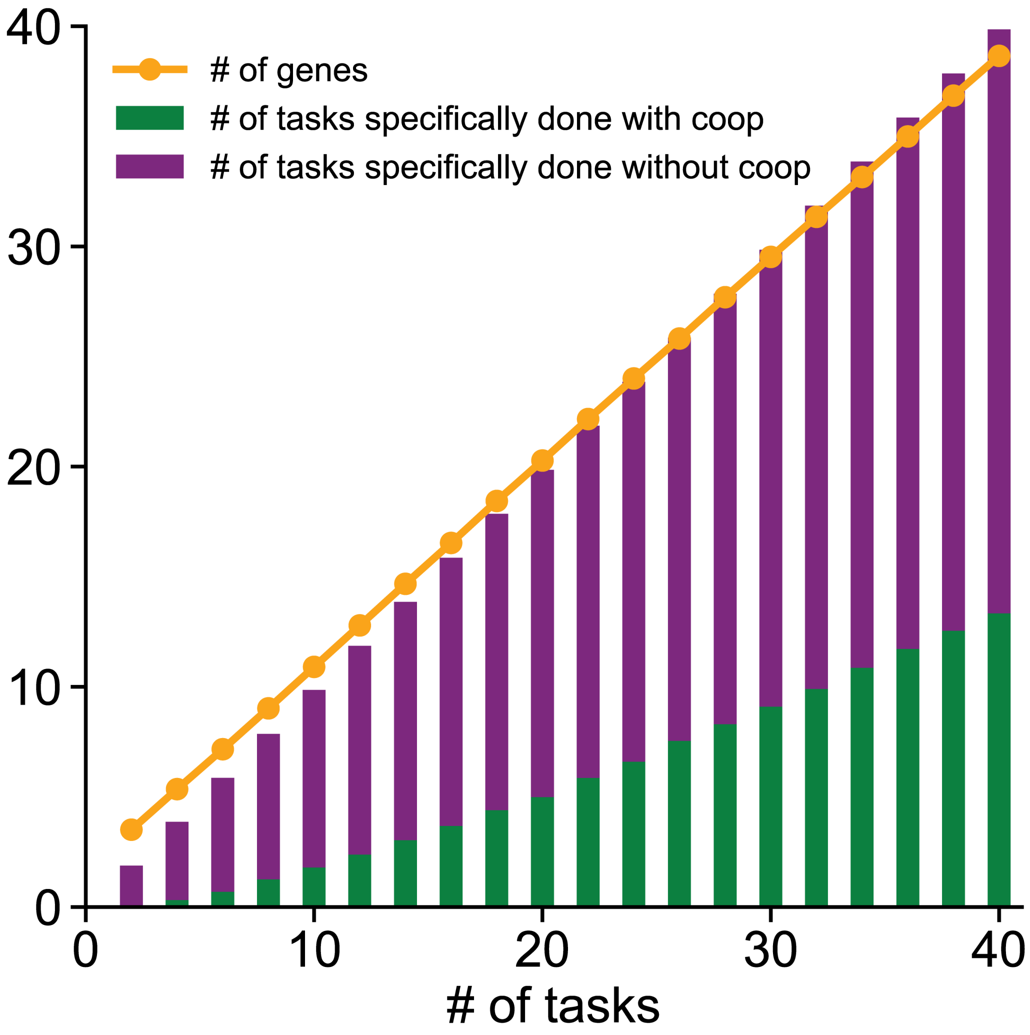

Fig. S9. The qualitative results discussed in the main text are robust to changing the number of characteristics required to describe the interaction from the value of three used to illustrate the results in the main text. Here, four characteristics are used to represent the characteristic space. ε_1 is changed to $0.3\varepsilon_2$ to make sure the ratio between the volume of the strong interaction regime (a 4D sphere of radius ε_1) and the volume of the weak interaction regime (a 4D sphere of radius ε_2) is the same as that of the reference system (the reference system is described by three characteristics). New tasks are introduced at a randomly chosen location that is $1.8\varepsilon_2$ away from any task from an earlier era, as was done for the reference system. The higher dimensionality of the characteristic space makes the new tasks less related to past ones compared to when three characteristics describe characteristics space. Therefore, weak cooperative interactions (WCI) evolve at a higher number of tasks. For example, when the number of tasks equals 40, 33% of tasks are done via WCI as shown in this figure, while this proportion is 56% for the reference system.

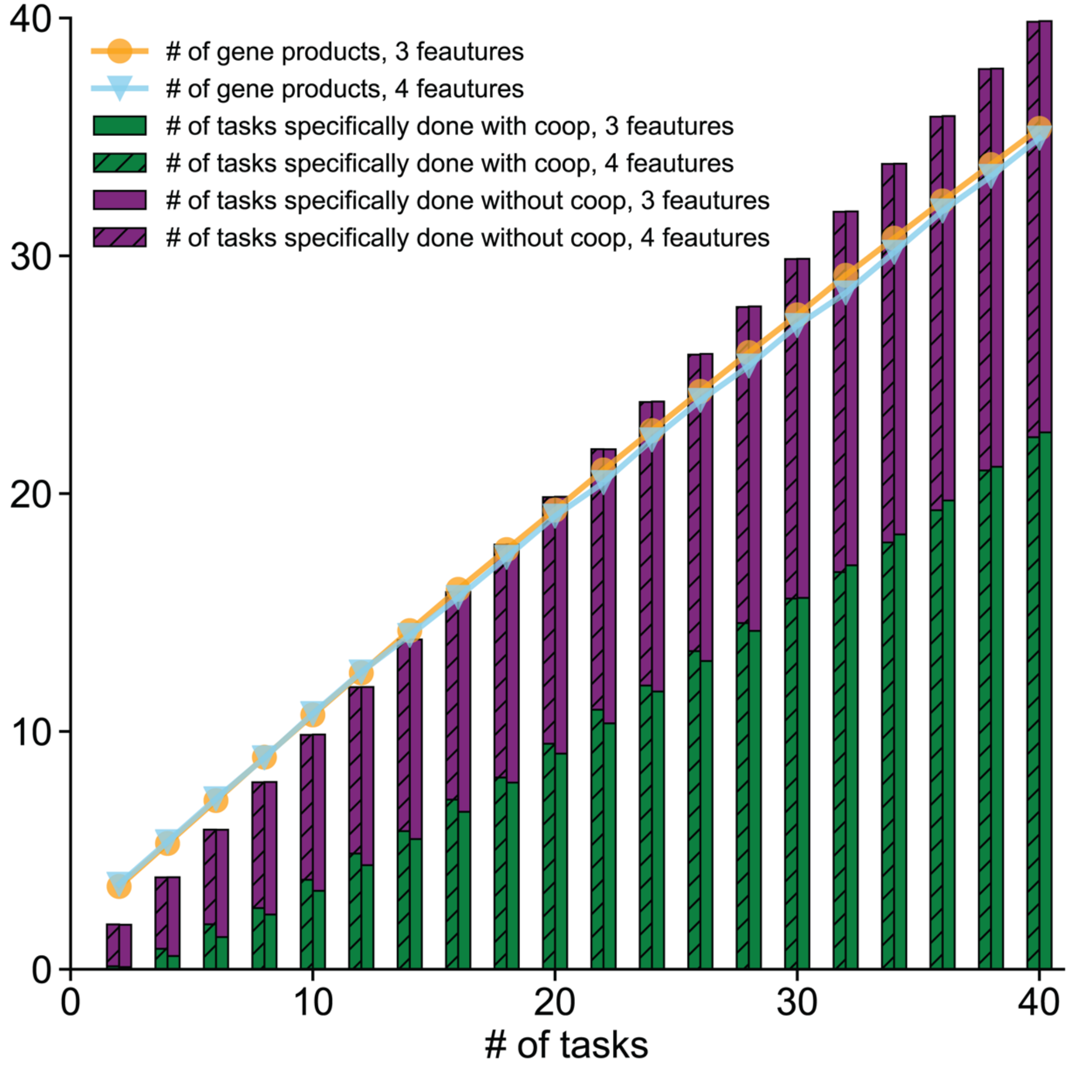

Fig. S10. Four characteristics are used to describe the characteristics space. New tasks are introduced at a closer distance ($1.4\varepsilon_2$) from a task from an earlier period (compared to when three characteristics suffice). The other parameters are the same as those used in Fig. S9. WCI evolve at a similar number of tasks compared to the reference system which is described by three characteristics.

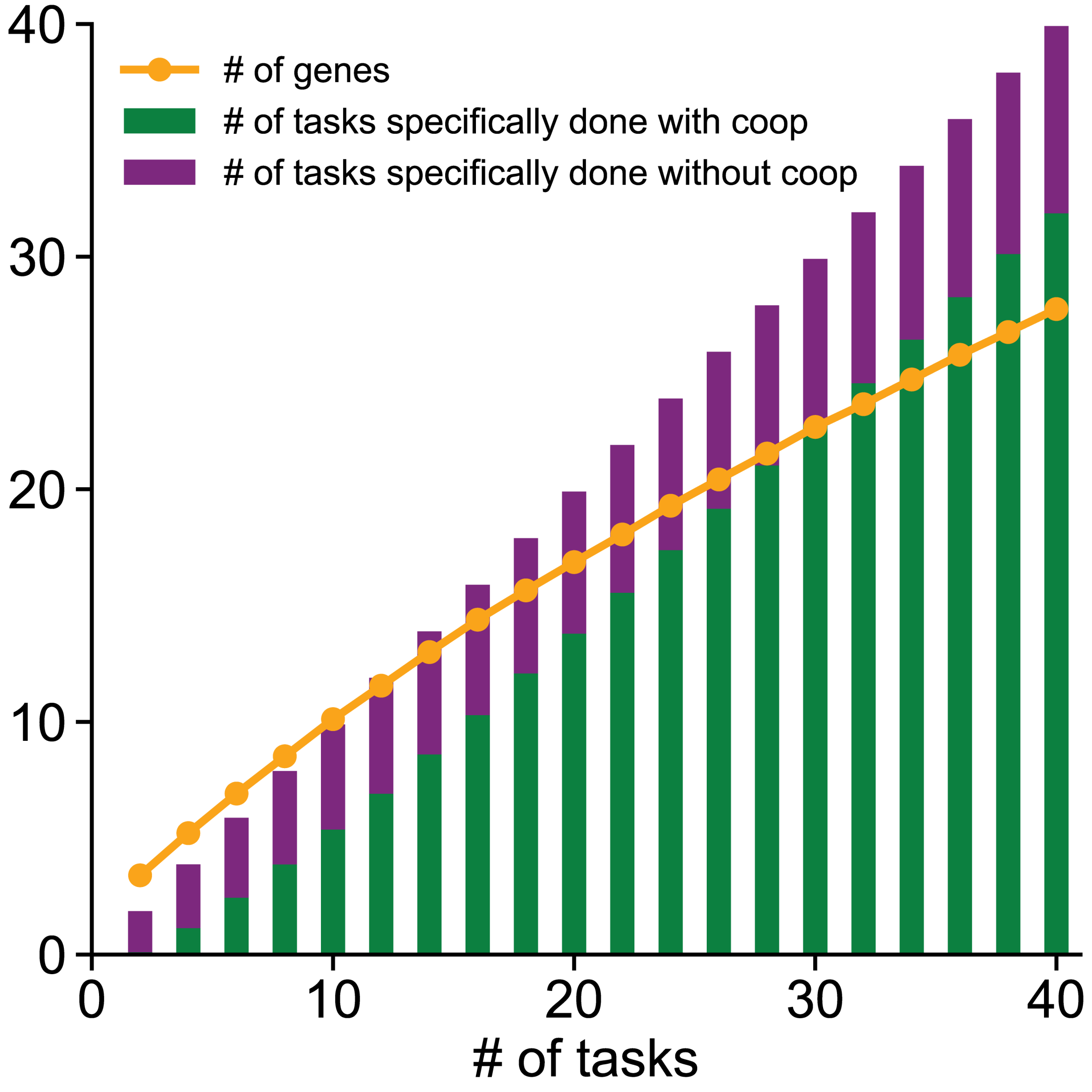

Fig. S11. The qualitative results discussed in the main text are robust to changing the number of characteristics required to describe the interaction from the value of three used to illustrate the results in the main text. Here, two characteristics are used to represent the characteristic space. ε_1 is changed to $0.09\varepsilon_2$ to make sure the ratio between the volume of the strong interaction regime (a 2D sphere of radius ε_1) and the volume of the weak interaction regime (a 2D sphere of radius ε_2) is the same as that of the reference system (the reference system is described by three characteristics). New tasks are introduced at a randomly chosen location that is $1.8\varepsilon_2$ away from any task from an earlier era, as was done for the reference system. The lower dimensionality of the characteristic space makes the new tasks more related to past ones compared to when three characteristics describe characteristics space. Therefore, WCI emerge at a smaller number of tasks. For example, when the number of tasks equals 40, 80% of tasks are done via WCI as shown in this figure, while this proportion is 56% for the reference system.
